# Pitfalls in Diagnosing Heterotopic Pregnancy in Sub-Saharan Africa: A Case Report at the Yaounde University Teaching Hospital (Cameroon)

**DOI:** 10.1155/2021/7970646

**Published:** 2021-11-18

**Authors:** Andre Ngandji Dipanda, Jovanny Tsuala Fouogue, Valere Koh Mve, Bruno Kenfack, Jean Dupont Ngowa Kemfang

**Affiliations:** ^1^Faculty of Medicine and Biomedical Sciences of the University of Yaounde 1, P.O. Box 1364, Yaounde, Cameroon; ^2^Faculty of Medicine and Pharmaceutical Sciences of the University of Dschang, P.O. Box 96, Dschang, Cameroon

## Abstract

Heterotopic pregnancy (HP) is a dizygotic twin pregnancy in which one gestational sac is intrauterine and the other is extrauterine. The prevalence of HP is unknown in Cameroon where the diagnosis is difficult and usually fortuitous like in other resource-poor settings. We herein depict pitfalls and delays in the diagnosis and management of a ruptured heterotopic pregnancy at the Yaounde University Teaching Hospital. After a wrong diagnosis and inadequate treatment, our patient presented at our emergency unit with severe pelvic pain and clinical signs of hemoperitoneum with shock. She underwent a total left salpingectomy through laparotomy. She had a complete spontaneous abortion five days after the surgery. Given that sonography is not routinely available in emergency departments in resource-poor settings, it may be relevant for practitioners to always bear HP in mind when facing ruptured ectopic pregnancies.

## 1. Introduction

Heterotopic pregnancy (HP) is a dizygotic twin pregnancy in which one gestational sac is intrauterine and the other is extrauterine, usually having tubal location. Since the report of the first case in 1761, the global prevalence has varied between 1/10000 and 1/30000 [1, 2]. The prevalence in Cameroon is unknown although some cases have been reported [3]. The diagnosis is very difficult when the clinical signs are those of a threatened abortion. Thus, in resource-poor settings where echography is not readily available, HP is frequently diagnosed fortuitously with higher morbidity and mortality than in rich countries. We herein depict pitfalls and delays in the management of a ruptured heterotopic pregnancy at the Yaounde University Teaching Hospital.

## 2. Case Presentation

**Figure 1:**
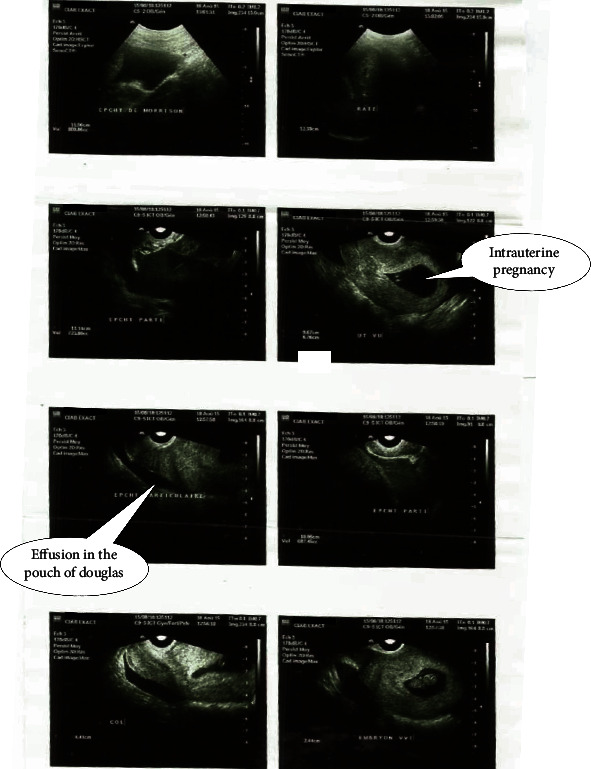
Ultrasound images showing intrauterine pregnancy and effusion in the Douglas pouch.

Mrs. M.F. (22-year-old, unemployed, Gravida 3, Para 1011) (history of one full term normal vaginal delivery and one termination of pregnancy during the first trimester) was admitted at the emergency unit at 8 weeks of pregnancy with severe pelvic pain of abrupt onset extending to the whole abdomen and radiating to the shoulder. The onset was 4 days prior to admission and there was neither trauma history nor vaginal bleeding. She fainted despite medication with paracetamol and a drip of amino acid pack administered by a community nurse at home to alleviate the pelvic pain and fatigue. She then went to a community health centre where ultrasonography revealed a 9-week-old intrauterine viable foetus with a massive peritoneal effusion (Figure 1). From there, she was referred to our emergency unit.

**Figure 2:**
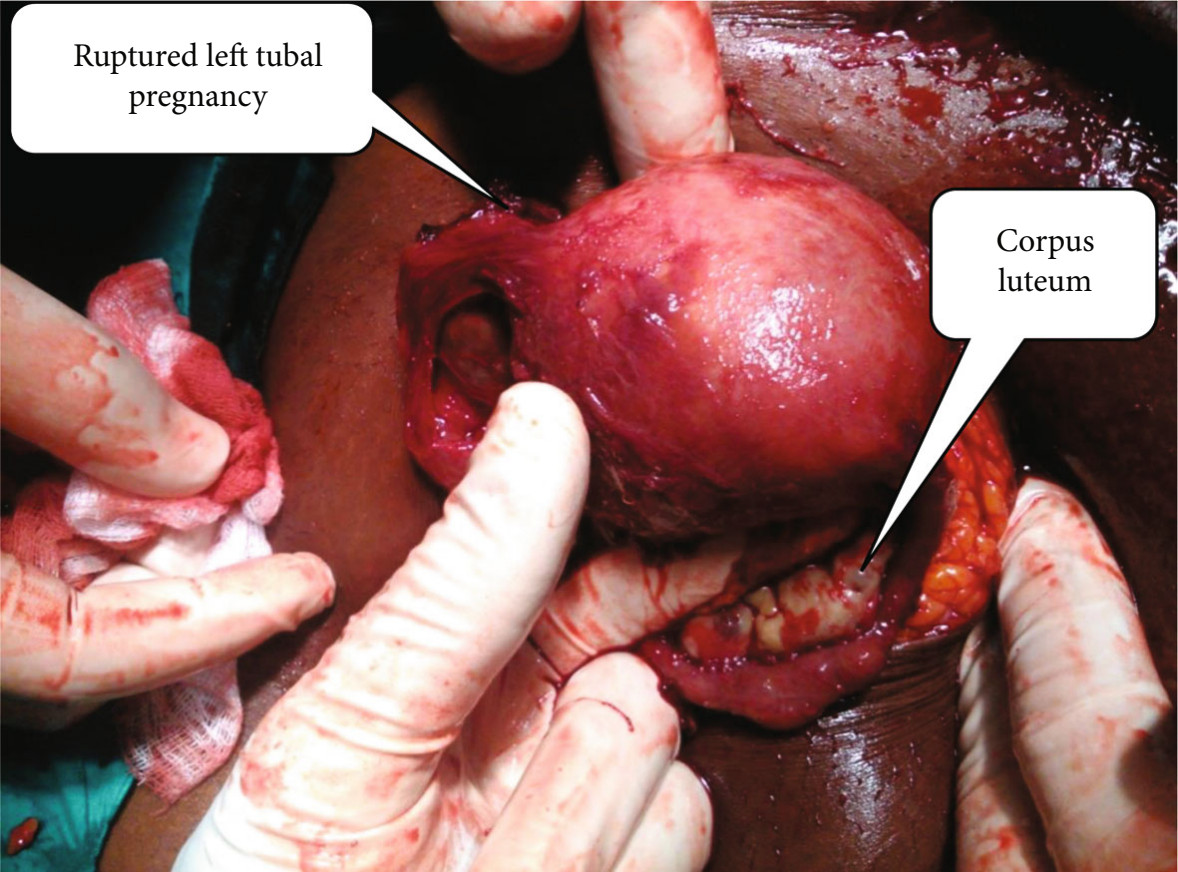
Intraoperative findings: ruptured left tubal pregnancy and contralateral corpus luteum.

Clinical assessment on admission revealed hemoperitoneum with shock (blood pressure: 72/56 mmHg; pulse rate: 120 pulsations/minute, respiratory rate: 28 cycles/minute, positive paracentesis). Our working diagnosis was a ruptured ectopic pregnancy with massive hemoperitoneum and shock. A very improbable associated intrauterine pregnancy was nevertheless evoked. The haemoglobin level was 6 grams/decilitre. Preoperative workup was unremarkable. The mainstay of management was emergency laparotomy under general anaesthesia with blood transfusion (only 1 pint of whole blood out of the 3 requested due to lack of means to afford). Laparoscopy was impossible because equipment was not available. Findings were as follows: hemoperitoneum (about 2000 millilitres), an increased and soft uterus (like 10 weeks of pregnancy), a ruptured left tubal (ampullary) pregnancy with contralateral corpus luteum, and bilateral tubo-ovarian adhesions (Figure 2). A total left salpingectomy was carried out followed by a cleansing of the abdominopelvic cavity. The uterus was handled very gently. Progesterone (7*α*-hydroxyprogesterone caproate) was administered prior to the surgery (1 gram via intramuscular route) and during the five postoperative days (500 milligrams/day via intramuscular route). Postoperative course was uneventful until day five during which she had an uneventful complete spontaneous abortion. The patient was discharged in a good condition 7 days after the surgery with iron supplementation (100 milligrams of ferrous sulphate twice a day for 3 months) because of a haemoglobin level of 8 grams per decilitre. She was seen 6 weeks later, and the attending physician found than she had fully recovered. During that visit, her opinion was asked and she complained of the late diagnosis by the nurse who attended her at home and of the expensiveness of management at the University Teaching Hospital. Six months later, she was still fine but had light periodic pelvic pains.

## 3. Discussion

The prevalence of HP is 1/30000 in the world, and very few cases have been reported in sub-Saharan Africa [3, 4]. Risk factors of HP combine those of ectopic pregnancy and those of multiple pregnancies. Our patient's medical history did not reveal such risk factors, but the presence of pelvic adhesions pointed a previous undiagnosed pelvic inflammatory process that favoured ectopic pregnancy. The diagnosis of HP is easy when signs of ectopic pregnancy are obvious, the most frequent being abdominopelvic pain, followed by other signs of peritoneal irritation [5].

The diagnosis is more difficult when signs of intrauterine pregnancy predominate with threatened abortion; it is also the case when voluntary termination of pregnancy without prior pelvic sonography is carried out (a common practice in resource poor settings): the endometrial mucosae and/or the intrauterine gestational sac is removed leaving the extrauterine one in situ. Our case illustrates pitfalls in diagnosis; indeed, she first received inappropriate care at home (painkillers and intravenous fluids). Proper management was done only at the stage of hemoperitoneum with shock.

Diagnosis of HP is made by ultrasonography with the following signs: an intrauterine gestational sac, an adnexal mass with hyperechogenic trophoblastic crown with or without embryo, and a possible presence of fluid in the pouch of Douglas [6]. In our case, echography was requested and done because symptoms and signs persisted. A systematic ultrasonography assessing the adnexal regions at the very beginning of pregnancy could have changed the course of events. Assessing adnexal regions the during first trimester ultrasonography examination is a must that should be strongly promoted in resource-poor context where task shifting allows nonradiologists and nongynaecologists to carry out the procedure.

Surgery (laparoscopy or laparotomy) is the main mean of management of HP [7]. The principle is to safeguard the intrauterine pregnancy and preserve fertility. We carried out an emergency laparotomy because of the massive hemoperitoneum, shock and lack of equipment and expertise for laparoscopy. Total left salpingectomy was carried out because the tube was much damaged.

Following surgery, 17.9% of women with HP will undergo spontaneous abortion [8]. Our patient had a spontaneous abortion five days after surgery. The possible causes are as follows: severe anaemia, prolonged exposure of the embryo to anaesthetic drugs, and handling of the uterus during surgery.

## 4. Conclusion

Heterotopic pregnancy is rare, and its diagnosis is difficult in our milieu where the first trimester ultrasonography is neither done routinely nor routinely available in emergencies. This case presented the morbidity due to late and inadequate management. It may be relevant for practitioners to always bear heterotopic pregnancy in mind when facing ruptured ectopic pregnancies.
